# Behavioral patterns of bats at a wind turbine confirm seasonality of fatality risk

**DOI:** 10.1002/ece3.7388

**Published:** 2021-03-18

**Authors:** Shifra Z. Goldenberg, Paul M. Cryan, Paulo Marcos Gorresen, Lee Jay Fingersh

**Affiliations:** ^1^ Conservation Ecology Center Smithsonian Conservation Biology Institute Front Royal VA USA; ^2^ Institute for Conservation Research San Diego Zoo Global Escondido CA USA; ^3^ U.S. Geological Survey (USGS) Fort Collins CO USA; ^4^ University of Hawaii at Hilo Hilo HI USA; ^5^ U.S. Geological Survey Pacific Island Ecosystems Science Center Hawaii Volcanoes National Park HI USA; ^6^ U.S. Department of Energy National Renewable Energy Laboratory National Wind Technology Center Boulder CO USA

**Keywords:** conservation behavior, ecological trap, migration, renewable energy, thermal infrared, video surveillance

## Abstract

Bat fatalities at wind energy facilities in North America are predominantly comprised of migratory, tree‐dependent species, but it is unclear why these bats are at higher risk. Factors influencing bat susceptibility to wind turbines might be revealed by temporal patterns in their behaviors around these dynamic landscape structures. In northern temperate zones, fatalities occur mostly from July through October, but whether this reflects seasonally variable behaviors, passage of migrants, or some combination of factors remains unknown. In this study, we examined video imagery spanning one year in the state of Colorado in the United States, to characterize patterns of seasonal and nightly variability in bat behavior at a wind turbine. We detected bats on 177 of 306 nights representing approximately 3,800 hr of video and > 2,000 discrete bat events. We observed bats approaching the turbine throughout the night across all months during which bats were observed. Two distinct seasonal peaks of bat activity occurred in July and September, representing 30% and 42% increases in discrete bat events from the preceding months June and August, respectively. Bats exhibited behaviors around the turbine that increased in both diversity and duration in July and September. The peaks in bat events were reflected in chasing and turbine approach behaviors. Many of the bat events involved multiple approaches to the turbine, including when bats were displaced through the air by moving blades. The seasonal and nightly patterns we observed were consistent with the possibility that wind turbines invoke investigative behaviors in bats in late summer and autumn coincident with migration and that bats may return and fly close to wind turbines even after experiencing potentially disruptive stimuli like moving blades. Our results point to the need for a deeper understanding of the seasonality, drivers, and characteristics of bat movement across spatial scales.

## Graphical abstract

Bat behavior driving fatalities at wind turbines remains poorly understood. Our analysis of one year of video imagery at a wind turbine in Colorado revealed seasonal peaks of bat activity that included increases in diversity and duration of behaviors that may exacerbate risk of fatality. Our results point to the need for a deeper understanding of the seasonality, drivers, and characteristics of bat movement across spatial scales.

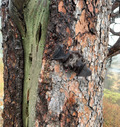

## 1 INTRODUCTION

**FIGURE 1:**
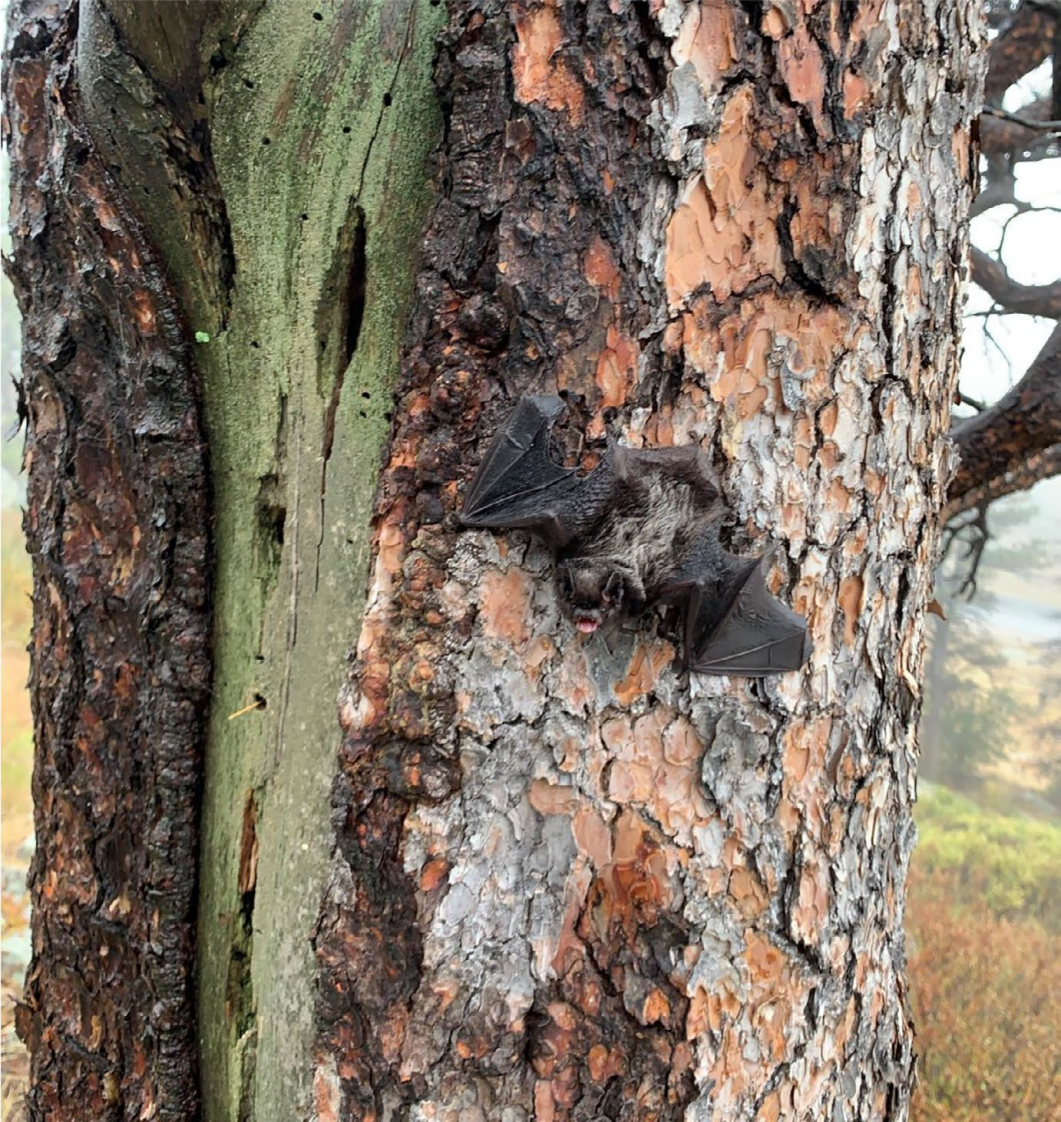
Migratory tree bats, like this silver‐haired bat (*Lasionycteris noctivagans*) seen roosting on a tree trunk during autumn, are among the most frequently found dead at wind turbines in North America during late summer and autumn

Wind energy is advancing as an environmentally clean alternative to fossil fuels that diversifies energy portfolios and creates new jobs (Gibson et al., 2017; Lindenberg et al., 2008). It has represented one of the fastest growing energy sectors in recent years, with over 90 countries incorporating wind energy by the end of 2016 (REN^21^, ^2017^). In the United States, wind represented 4.5% of the country's annual electricity production at the end of 2013 and may feasibly reach 20% by the year 2030 and 35% by 2050 (Lindenberg et al., 2008; USDOE, 2015). However, wind energy has been associated with wildlife fatality as birds and bats collide with turbine blades, the tips of which can spin faster than 50 m/s. These impacts are likely to intensify as wind development continues (Gibson et al., 2017; Kunz et al., 2007; Northrup & Wittemyer, 2013; O’Shea et al., 2016). Avoiding turbine placement along flyways and within identifiable preferred habitat has emerged as a viable mitigation strategy for birds, yet site selection of turbines may not have a similar benefit for bats as bats may be attracted to turbines (Arnett & May, 2016; Mojica et al., 2016). At present, curtailment of turbine blades during specified weather conditions is one promising mitigation strategy (Arnett et al., 2011; Arnett & May, 2016), but is a coarse approach that may be further refined with a better understanding of bat behavior at turbines. Insectivorous bats play important ecological roles and provide critical ecosystem services (Kunz et al., 2011). The possibility that wind turbines may act as population sinks for bats is therefore of considerable conservation concern (Cryan & Barclay, 2009), especially given their slow life history (e.g., long‐lived with few, slow‐maturing offspring; Barclay & Harder, 2003) and evidence that wind turbines are among the most prominent threats to the well‐being of certain bat populations (Frick et al., 2017; O’Shea et al., 2016). Although fatalities of many bat species have been found at wind turbines in temperate parts of the United States (USA) and Canada (Grodsky et al., 2012; Jain et al., 2011), the vast majority (>75%) involve three species that are ecologically similar: the hoary bat (*Lasiurus cinereus*), eastern red bat (*Lasiurus borealis*), and silver‐haired bat (*Lasionycteris noctivagans*) (Arnett et al., 2008; Frick et al., 2017) (Figure 1). These species are unique among North American bat species in being almost exclusively dependent on trees for roosting, continental in their distribution, and migratory. Therefore, it is possible that aspects of their life history make them more susceptible to turbine collisions than other species.

Another clear pattern in temperate parts of North America is that most turbine bat fatalities occur from July through October, usually peaking in August or September (Arnett & Baerwald, 2013; Cryan & Brown, 2007). Patterns of turbine‐related bat mortality in Europe show a similar temporal pattern but often involve a greater diversity of species in terms of both migratory behavior and roosting ecology (Rydell et al., 2010a; Voigt et al., 2012, 2015). Video imagery has revealed bats closely approaching and exhibiting unexplained behaviors at turbine blades, nacelles (housing that holds turbine machinery at top of structure), and monopoles (cylindrical steel tower supporting nacelle and blades), as well as repeatedly approaching turbines after near contact with moving blades (Cryan, Gorresen, et al., 2014; Horn et al., 2008). Despite the pattern of most North American bat fatalities occurring at wind turbines in autumn and involving migratory, tree‐dependent species, it is not known whether mortality patterns are attributable to seasonal bat prevalence at wind turbines, temporally variable investigative behaviors, or some combination thereof. Determining whether behaviors at wind turbines are seasonal and discovering any underlying causes of bat investigation are promising paths toward enhancing concrete, evidence‐based recommendations for effectively mitigating the impacts of wind energy on bat populations (Cryan & Barclay, 2009; Jameson & Willis, 2014). Analyses of the behavior of bats at wind turbines offer a unique opportunity to better understand bat susceptibility to this emerging technology. In this study, we used thermal video imagery from a wind turbine continuously monitored over a year‐long period in Colorado, USA, to describe temporal trends in the behaviors of bats. We discuss our results in the context of potential bat attraction to turbines and knowledge gaps in bat ecology.

## 2 MATERIALS AND METHODS

### 2.1 Data collection

**FIGURE 2:**
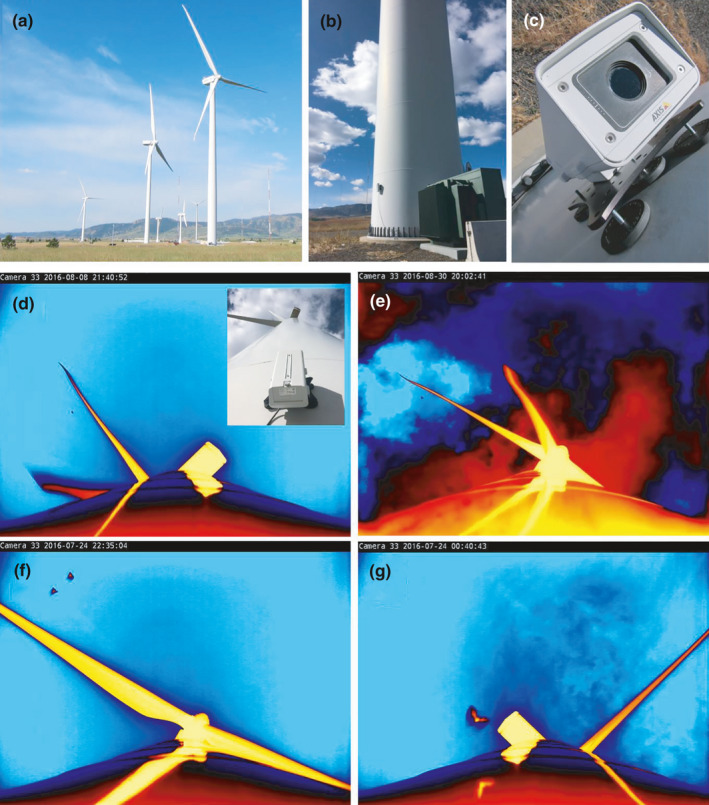
Bats were observed over a one‐year period (17 March 2016 to 16 March 2017) at a wind turbine in Colorado with a thermal‐spectrum video camera (a). The camera was magnetically mounted on the turbine monopole approximately 2 m above the ground (b) and pointing upward (c). By pointing toward the turbine nacelle and rotor‐swept area (d, inset), the camera recorded bats as they flew at rotor‐swept heights (approx. 42–119 m; d, e), as well as bats flying closer to the ground (f, g)

We recorded video imagery on a near‐nightly basis over one year from a wind turbine at the National Wind Technology Center, National Renewable Energy Laboratory in Boulder, Colorado. We selected this turbine for the study because it was conveniently located and made available to us for year‐round observation and maintenance access. The 1.5‐MW wind turbine (39.9121°N, 105.2200°W; Model GE 1.5sle, General Electric Renewable Energy, Schenectady, New York, USA) had a tapered monopole that was 80 m tall and 4.2 m in diameter at the base. The nacelle at the top of the monopole housed the generator and turbine blades with a 77‐m rotor diameter. The turbine was surrounded by urbanized and arid rangeland transected by several drainages to the north, east, and south, and transitioning into foothills of the Rocky Mountains approximately 5 km to the east (Figure 2a). Five additional wind turbines of various makes and sizes also operated in an approximate line running 1 km to the southwest. Historical and current weather conditions at the site, measured at approximately 1.25 km WSW of the turbine, are available at https://midcdmz.nrel.gov/apps/go2url.pl?site=NWTC.

We monitored airspace swept by the rotors of the turbine using a surveillance camera equipped with a 19‐mm lens (Axis Q1932‐E, Axis Communications, Lund, Sweden) that imaged in the thermal infrared spectrum (~9,000–14,000 μm) of electromagnetic radiation. The camera was operated at a sampling rate of 30 frames per second (fps), a resolution of 640 by 480 pixels, and in the built‐in false‐color scheme “Ice‐and‐Fire” with no supplemental illumination. We magnetically mounted the camera approximately 2 m above ground level on the east side of the turbine monopole using an industrial‐strength camera mounting base (RigMount X6 Magnet Camera Mounting Platform, Rigwheels, Minneapolis, Minnesota, USA; Figure 2b,c). The camera was aimed straight up the monopole so that the lower third of the video image included the monopole, while the upper two‐thirds of the image included the turbine blades, nacelle, and surrounding airspace, encompassing approximately 0.30 km^3^ of airspace from a field of view 86.6 m wide by 64.2 m high at a range of 160 m (Figure 2d, inset). This configuration allowed us to observe the behavior of bats as they approached the leeward side of the turbine (prevailing wind direction was from the west) at various heights above the ground (Figure 2d–g). This monopole‐mounted configuration also provided an ideal view of the bat's horizontal proximity to the turbine relative to prior video configurations (e.g., Cryan, Gorresen, et al., 2014; Horn et al., 2008). The camera view became obscured after precipitation events, but usually cleared within a few hours after precipitation ceased due to the heated window on the weatherproof housing. Video monitoring for this study began on 03 March 2016 and continued through 17 March 2017. The video camera was programmed to record each night from 18:30 to 07:00 the following morning (Denver local time) between 03 March and 20 July of 2016, from 19:00 to 07:00 between 21 July and 26 October of 2016, and then 17:00 to 07:00 from 27 October 2016 through 17 March 2017. This shifting schedule ensured that the camera consistently recorded during twilight hours throughout the year. The camera was powered through a single cable using a power‐over‐ethernet (POE) network switch (Model GS105E, NetGear, San Jose, CA, USA) and communicated through the same cable with a laptop computer (Model Latitude E5430, Dell Inc., Round Rock, TX, USA) situated just inside the door in the base of the monopole. Video recording software included with the camera (Axis Camera Station 5.x, Axis Communications, Lund, Sweden) was used to export imagery buffered on the computer's hard drive to a 1‐ to 2‐TB external hard drive (Backup Plus Slim, Seagate Technology LLC, Cupertino, CA, USA) after recording ended each morning. These nightly video files were containerized into Advanced Systems Format (.asf) using the H.264 compression codec, then converted into audio‐video interleave (.avi) container format using a GNU General Public License video editing program (VirtualDub, http://www.virtualdub.org/) prior to analysis.

### 2.2 Data analysis

We analyzed imagery spanning one year, from 17 March 2016 through 16 March 2017. Of the 365 nights during which we attempted to record imagery, recording failed on 49 nights (13%). Gaps in recording were primarily spread over the off‐season (December, January, February, and early March), with the exception of two consecutive nights in August and three consecutive nights in September. Recordings on 13 nights (4%) were incomplete (<10 hr of imagery) due to technical issues, and imagery recorded on 10 nights (3%) was obscured by precipitation. Incomplete nights were distributed across the study period, with a cluster of four incomplete nights over the course of a week in April, two consecutive nights in June, two consecutive nights in July, and the remaining five incomplete nights scattered across months of low bat occurrence. Eight of the 10 nights obscured by precipitation were clustered as 3 and 5 consecutive nights in April. We thus recorded approximately 3,800 hr of analyzable imagery over the course of 306 nights. Videos were loaded into the program MATLAB with the Image Processing Toolbox (versions 2015a,b, MathWorks, Inc., Matick, MA) using previously developed custom code (Cryan, Gorresen, et al., 2014). The algorithm detected videos containing bat‐sized objects not associated with the visual footprint of the turbine moving through the field of view. We manually reviewed these videos (hereafter “detections”), categorizing them as “bat,” “bird,” or “insect” and assessed confidence that the object was a bat by categorizing them as “high,” “medium,” or “low” based on bat appearance and movement characteristics (Huzzen et al., 2020). Only high‐confidence bat detections (hereafter “bats”) were included in analyses. For consistency, this categorization was done by one primary observer (SZG), and then, all high‐confidence bats were reviewed and cross‐validated by a secondary observer (PMC). An “event” was temporally defined as any string of detections occurring one minute or less apart, such that if bats went out of view they were not counted as independent events if they reappeared within one minute or less; this is consistent with previous work by Cryan, Gorresen, et al., (2014). Events were comprised of one or more activities that characterized the approach location (monopole, nacelle, blade), flight type (nonfocal pass (sensu Cryan, Gorresen, et al., 2014; Huzzen et al., 2020), hovering, chase involving at least one other bat), and outcome (displacement or possible strike by turbine blade) of the detection (Table 1). We defined a displacement as any event during which a bat was visibly moved through the air after it passed within approximately 5 m of a moving turbine blade, but during which there was no visible contact between the bat and the turbine blade. Unambiguous contact between flying animals and moving turbine blades is difficult to determine in thermal imagery, and because we did not conduct concurrent ground searches for bat fatalities around the wind turbine, the events in which a moving blade appeared to make physical contact with a bat are hereafter referred to as “possible strikes.”

**TABLE 1:** Definition of observed bat activity grouped by outcome (displacement, possible strike), flight behavior (chase, nonfocal pass, hovering), and turbine location (blade, monopole, nacelle)

	Activity	Definition
Outcome	Displacement	Bat appears to be moved by turbine blade
	Possible strike	Bat appears to be struck by blade and falls
Flight behavior	Chase	Close following flight involving at least two bats
	Nonfocal pass	Pass by turbine air space without interaction
	Hovering	Persistent nondirectional flight in same location
Turbine location	Blade approach	Flight path directed toward blade
	Monopole approach	Flight path directed toward monopole
	Nacelle approach	Flight path directed toward nacelle

To determine temporal trends in bat behavior, we used negative binomial regression models with nightly tallies of each activity as response variables. We included the day of the bat season as a predictor variable and defined bat season as beginning on the night of the first bat detection and ending on the night of the last bat detection. To control for irregular occurrence of bats throughout the year, we included the log of the total number of bat events recorded for that night as an offset in models. Regression models were run using the package glmmTMB (Magnusson et al., 2017) and checked for overdispersion using the package DHARMa (Hartig, 2020) in R (R Development Core Team, 2010).

## 3 RESULTS

We detected bats during 177 of the 306 nights analyzed. The earliest date on which bats were detected at the wind turbine was 04 April 2016 and the last bat detection occurred 19 November 2016 (Figure 2). Bat events gradually increased throughout the spring, summer, and early autumn with the highest number of bat events occurring in September (*n* = 748), July (*n* = 563), and August (*n* = 528). The median number of bat events per night (excluding nights when no bats were detected) was 11 (interquartile range: 4–21). All high activity nights (nights above the interquartile range with > 21 events per night) occurred between 04 June and 08 October, with the highest number of single‐night events (92) occurring on 02 October.

**FIGURE 3:**
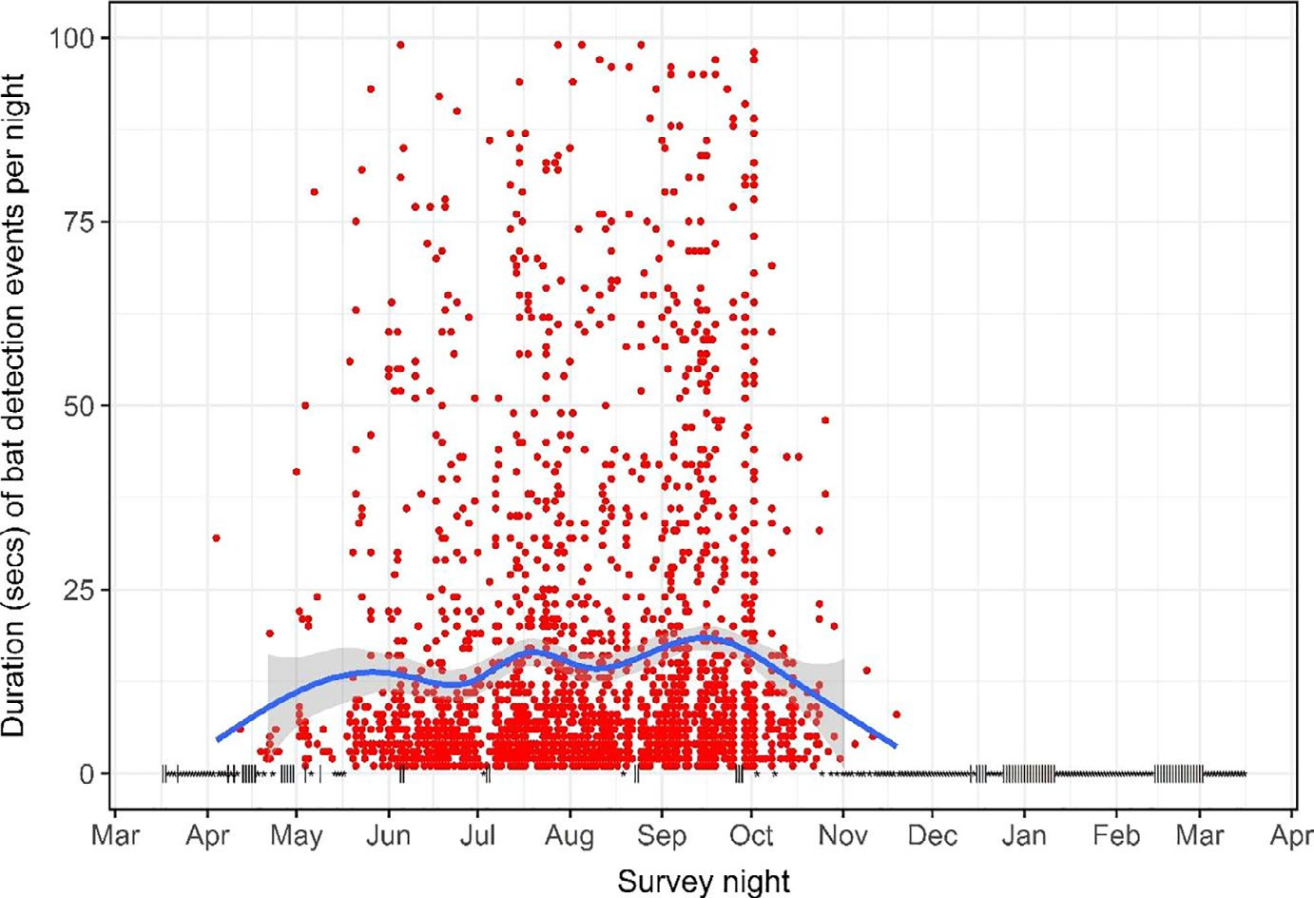
Duration (seconds) of individual bat events (red points) by date. Loess curve and 95% CI (shading) highlight change in duration over time, and y‐axis is truncated at 100 s to better depict seasonal pattern. Black points and vertical dash symbols depict sampled nights with no detections and unsampled nights, respectively

**FIGURE 4:**
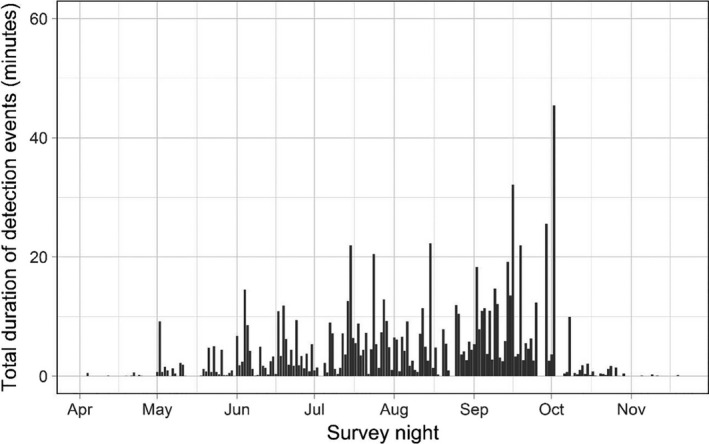
Total duration (minutes) of all bat events (i.e., detections that are connected by 1‐min or less) by survey night

**FIGURE 5:**
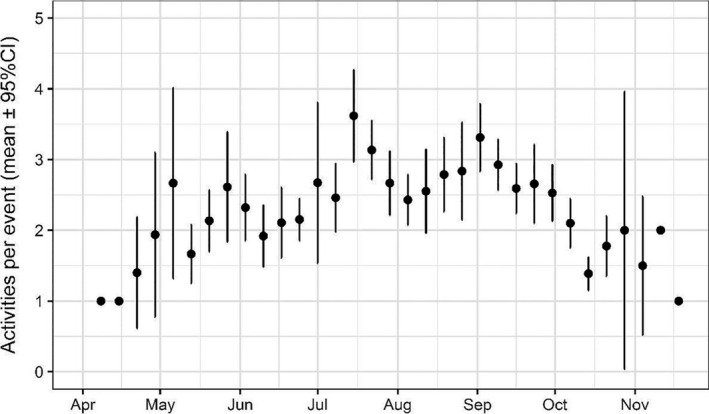
Number of nightly observed activities per detection event per week (mean with 95% confidence intervals)

**FIGURE 6:**
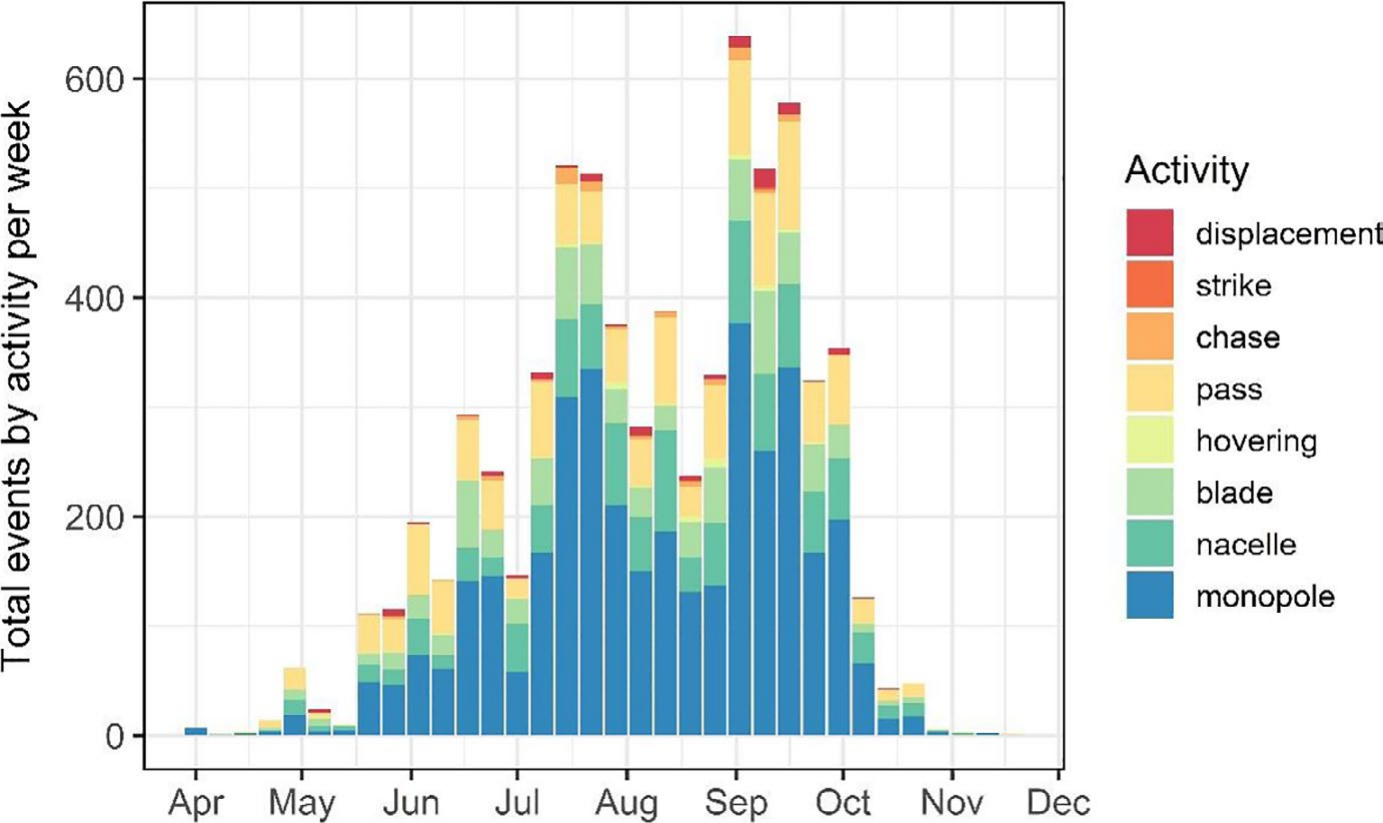
Observed bat activity by activity type and date. See Table 1 for definitions. Activities exhibited two distinct seasonal peaks in July and September, which were largely driven by approaches to all parts of the turbine

**FIGURE 7:**
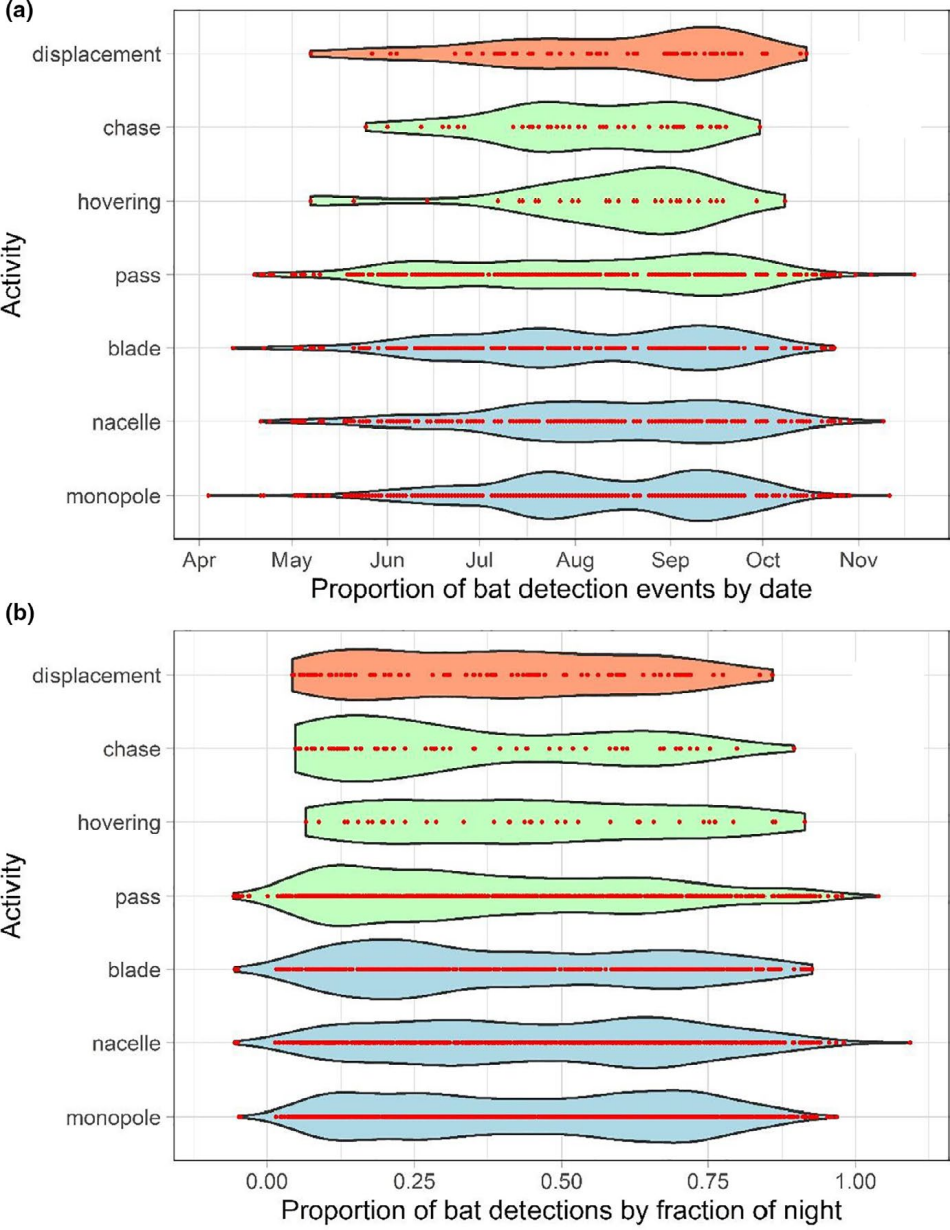
Seasonal (a) and nightly (b) trends in the timing of activities and interaction outcomes of bats observed. Observations are grouped by outcome (displacement), flight behavior (chase, hovering, nonfocal pass), and turbine structural location of close approaches (blade, nacelle, monopole). Violin plots depict density of observations by width of plot bar. Points (red) indicate distinct events. Each state's bar has an equal area to make comparable the density of distributions over time

Only two possible strikes were detected; these activities were pooled with displacements for subsequent analyses. The duration of events and the number of discrete activities observed per event differed over the course of the season (Figures 3, 4, 5). Bats exhibited longer events at turbines between July‐September compared to other months: the mean number of discrete activities per event for these months (July: 3.03, August: 2.58, September: 2.87) was higher than the 95% confidence intervals of all other months during which bats were recorded (Figures 3, 4). The number of activities observed per event seasonally peaked in July and September, with a significant decline in August (the mean in August was below the 95% confidence intervals of July and September) (Figures 5, 6). Approaches to all components of the turbine were most frequent in July and September, as was chasing flight involving two or more bats (Figures 6, 7a). Other bat activity (nonfocal passes and hovering flight) and the outcome of turbine interactions (displacements) peaked in mid‐September (Figure 7a). However, when the incidence of each activity was analyzed while controlling for the total number of bat events on a given night in generalized linear models, nonfocal passes were negatively related to day of the season (β (SE) = −0.002 (0.001), *p* = .021), whereas monopole and nacelle approaches were positively related to day of the season (monopole: β (SE) = 0.002 (0.001), *p* = .017; nacelle: β (SE) = 0.003 (0.001), *p* = .018). Blade approaches, displacements, hovering flight, and chases were not significantly related to date, likely due to the relative infrequency of these events.

Bats being displaced by turbine blades were observed 104 times during 79 discrete events (Table 2). Many of these displacement events (58%) involved a separate turbine approach behavior prior to the displacement (i.e., within the same bat event). Bats returned to approach turbines following displacement in about half (51%) of the observed events that did not end in possible strikes (*n* = 77), 35 of which involved bats that never left the field of view and were therefore certainly the same bat returning to the turbine after displacement. Multiple displacements during a single event were recorded during 22% of the events that did not end prematurely due to a possible strike. Most of these multiple displacement events (65%) occurred in September.

**TABLE 2:** Number of nightly bat activities (*n* = 6,987) observed for all bat detection events (*n* = 2,656) recorded from 17 March 2016 to 16 March 2017

Activity	Total	Mean	Max
Displacement	104	1.32	4
Possible strike	2	1.00	1
Chase	83	1.41	4
Nonfocal pass	1,198	1.28	10
Hovering	48	1.26	3
Blade approach	794	1.50	10
Monopole appr	3,679	2.26	22
Nacelle appr	1,079	1.66	22

Most bat detections occurred during the first half of the night, and activities and interaction outcomes also exhibited trends throughout the night (Figure 7b). The pattern of proportionally more detections early in the night was especially pronounced for blade approaches, chase behavior, and nonfocal passes, which were concentrated near dusk. Activities involving close approaches to stationary parts of the upper wind turbine, such as the nacelle and monopole, showed bimodal proportional increases after dusk and then again in the early morning hours (approximately 01:00–03:00). Hovering flight and observations of displacements were more dispersed throughout the night than other types of observations.

## 4 DISCUSSION

Given the lack of understanding about behaviors of migratory bats in general, assessing seasonality of their interactions with wind turbines offers valuable information toward understanding fatalities—a distinctly seasonal phenomenon. This is particularly true if wind turbines are being perceived as trees by migrating bats and attracting them to landscapes with turbines regardless of whether bats were common in the area prior to wind facility siting. If such attraction is occurring, wind turbines might be acting as ecological traps (poor‐quality habitat that animals visit after following environmental cues generally associated with natural favorable habitats (Schlaepfer et al., 2002)).

Our findings from this observational study are consistent with previously reported trends from other wind energy facilities in temperate North America and Europe indicating that bat activity near wind turbines increases in the later months of summer and into autumn (Arnett & Baerwald, 2013; Cryan & Brown, 2007; Rydell et al., 2010b). The number of bat detection events we observed, as well as the number of times bats repeatedly approached turbines within events, increased as the season progressed. Further, most events involving the highest risk behavior (multiple displacements of bats by turbine blades) occurred in September, which has consistently been a peak mortality month for bats at wind turbines in North America (Arnett & Baerwald, 2013). Together these results show seasonal variability in the behavior of bats in proximity to wind turbines and support the notion that the risk of turbine‐related bat mortality increases as summer progresses to autumn.

Bat activity during the recorded year was highest at the turbine in July and September, in terms of both the number and length of bat events (Figures 4, 5). The increased duration of bat events at the turbine appeared to be primarily driven by close‐approach behaviors. Indeed, these investigative behaviors were positively related to day of the season whereas passing through the airspace without investigation (“nonfocal pass”) was negatively related to the day of the season when controlling for the number of events in a night, indicating a seasonal shift in the activities bats exhibited. The high prevalence of behaviors associated with collision risk, such as repeated approaches close to turbine surfaces and the increased duration of time spent at turbines during times bats were making close approaches, lends further support to the idea that bats exhibit seasonal investigative behavior at wind turbines. These behavioral patterns are consistent with observations from earlier video‐based studies (Cryan, Gorresen, et al., 2014; Horn et al., 2008), yet our new year‐long study confirms the previously assumed seasonality of risky behaviors of bats at turbines. Given the association between risky behaviors and the migration season for bats, these results further point to the need for a deeper understanding of migratory processes in bats and how they may differ among years, sites, and species.

Visual observations of bats at wind turbines thus far indicate that bats often seek some type of resource around these tall landscape structures. Several potentially perceived resources are suspected of attracting bats to wind turbines, including insect prey, roosts, and mating opportunities (Cryan, 2008; Cryan & Barclay, 2009; Horn et al., 2008; Kunz et al., 2007; Rydell et al., 2010b; Rydell, 2016). The seasonally variable behaviors bats exhibit at wind turbines could be influenced by multiple underlying factors, including the onset of mating, hyperphagia driven by late‐summer concentrations of insects, the need to accumulate fat for hibernation and migration, or simply required rest stops and shelter during migration. If bats obtain the resources they seek at turbines, the presence of such resources (e.g., insect concentrations or other bats) or utilization of any resources present (e.g., successfully roosting on turbines or regularly foraging) should be observable.

There is indirect evidence that bats feed in the minutes to hours before fatally colliding with turbines (Bennett et al., 2017; Foo et al., 2017; Reimer et al., 2010; Rydell, 2016; Valdez & Cryan, 2013), and indications from feces in door slats and transformer gills that bats may roost on turbine features during nightly foraging bouts (Bennett et al., 2017; Foo et al., 2017). The behaviors we observed around wind turbines could not be attributed solely to feeding, nor did we see bats landing and roosting on turbine surfaces. Thus, while bats may be roosting on or feeding near turbines in certain situations, these activities were not obvious to us. Most bats dying at wind turbines tend to involve species thought to feed primarily “on the wing” and that are morphologically adapted to an aerial‐hawking feeding strategy (e.g., long, narrow wings; Norberg & Rayner, 1987). The gastrointestinal contents of bat carcasses found beneath wind turbines sometimes contain potentially nonflying insect forms, suggesting bats might sometimes glean prey from turbine surfaces (Reimer et al., 2010; Cryan and Valdez, 2013). It is possible that the close approaches we frequently observe bats making to wind turbines during late summer and autumn involve attempts to glean insects from turbine surfaces (e.g., Rydell, 2016; Valdez & Cryan, 2013). We observed no instances of bats actually gleaning insects, which were often present on turbine surfaces within view of the camera, in the hundreds of hours of video we analyzed for this study. We remain skeptical that the close and risky approaches bats frequently make to wind turbine surfaces in late summer and autumn are exclusively driven by foraging attempts (Reimer et al., 2018), although comparison with other turbine types and sites is warranted.

If bats approach wind turbines with an expectation of resources that is not met (e.g., they are seeking suitable roosts and do not find them), they may move on rather quickly once they learn that the resource is not available (Cryan, Gorresen, et al., 2014). A plausible explanation for why we observed bats closely approaching the turbine monopole during July and September more frequently than any other seasonal activity is that they simply mistook it for the trunk of a tree. It is possible that the waves of bat activity and close‐approach behaviors we observed at the turbine in July and September, and August to a lesser extent, were attributable to passing migrants investigating unfamiliar structures, and therefore increased abundance of bats in the area investigating potential resources during those seasonal peaks. The sudden decline in bat activity following the early October peak is also consistent with the possibility of migration waves of nonresidents driving turbine behaviors. Hoary bats and silver‐haired bats migrate through Colorado, perhaps at different times, and their presence in the area around the turbine is expected to vary with season (Cryan, 2003). For example, at a wind facility in Alberta, Canada, peak fatality numbers of hoary and silver‐haired bats found beneath wind turbines during the day corresponded to the temporal pattern of echolocation calls detected at night—these species‐specific peaks differed by a few weeks, indicating that silver‐haired bats were migrating through the area later than hoary bats (Baerwald & Barclay, 2011). We did not differentiate bats by species, so it is unclear whether observed patterns are indicative of species‐specific movement trends. Future research that pairs information on bat species composition at turbines with species‐specific seasonal behavioral patterns may provide unique insight, as the resources sought or found at turbines may differ across species, seasons, years, and sites (Bennett et al., 2017; Foo et al., 2017). The extent to which bats track migratory insects (Hu et al., 2016; Rydell et al., 2010b; Satterfield et al., 2020) may also be an important factor in untangling these seasonal patterns.

Temporal patterns in bat activity and behavior that we observed during the nights at the wind turbine may also hint at the origins of the bats involved. Although bats tended to visit turbines more often before midnight, most behaviors were scattered throughout the night. If bats we observed resided near the turbine and were familiar with the area, we would have expected activity to be associated with foraging, which typically peaks in bats during the early part of the night, followed by cessation of foraging activity near the middle of the night (Erkert, 1982). Such a pattern was not apparent in our observations. We cannot rule out the possibility that a proportion of our observations involved local resident bats, some of which might belong to the same species that also seasonally migrate through the area in larger numbers. Future studies that integrate acoustic detectors with tracking devices could help determine whether migratory bats are more abundant or engaging in some type of seasonally variable behavior that places them at higher risk than nonmigratory, resident bats. To this end, determining differences in behavioral patterns across or within species could be illuminating. Studies in other taxa have revealed considerable differences in movement strategies among individuals within the same species. For example, while they partially overlap in space, resident and transient orcas (*Orcinus orca*) specialize in different prey species leading to resource tracking over much broader ranges in transient as compared to resident orcas (Andrews et al., 2008). The determinants of residency versus migration in eastern red, hoary, and silver‐haired bats, and whether these bats are more vulnerable at wind turbines if they are not resident, remain key questions.

Neither the extent to which bats involved in wind turbine fatalities exhibit range residency, nor the routes taken by individuals that actually make large‐scale movements are well understood (Fleming, 2019). GPS tracking data across numerous taxa, including bats, has allowed for clearer categorization of movement patterns (Abrahms et al., 2017; Roeleke et al., 2016; Weller et al., 2016). These studies have implications for better understanding the proportion of individuals comprising a population that make long‐distance movements, as well as the resources that animals track and revisit over time. Whether eastern red, hoary, and silver‐haired bats exhibit nomadism, characterized by limited site fidelity across years or migration, characterized by high interannual site fidelity, has implications for the risk that turbines pose to individuals that encounter them. The findings presented here highlight the many knowledge gaps that remain in bat migration ecology. Narrowing these gaps may be highly beneficial to developing effective mitigation strategies at wind facilities. We recommend future research address the drivers of migratory bat movement at different spatial scales.

Our results should be interpreted with caution given our focus on a single turbine at a single site, yet the high temporal and behavioral resolution of data presented here sheds new light on bat behavior at wind turbines while highlighting potential future research directions. Reliance on video‐based studies such as ours has only revealed behaviors of bats at wind turbines (within approximately 50 m) (e.g., Cryan, Gorresen, et al., 2014; Horn et al., 2008; Huzzen et al., 2020). Experiments to determine whether and how wind turbines seasonally attract bats from farther distances and at relevant landscape scales in North America have not been published but are critical for elucidating the concept of resource selection along migratory routes and determining whether turbine design or siting criteria could help mitigate the risk to bats. Such studies could also help inform the potential of deterrents (such as sound or light‐based deterrence devices) to reduce bat mortality. However, our documentation of repeated displacements, if they are also perceived by the bats as aversive stimuli, raises concerns for the efficacy of these mitigation measures. Our observations of repeated interactions of bats after being physically displaced by the turbine blades emphasize the importance of identifying the behavioral motivations of bats within the rotor‐swept zone. The thermal video surveillance and behavioral analysis approach developed for this study represents a practical and robust way to quantify these interactions and may help guide the development of strategies that reduce bat fatalities.

## CONFLICT OF INTEREST

We declare no competing interests.

## AUTHOR CONTRIBUTIONS

**Shifra Goldenberg:** Conceptualization (equal); Data curation (equal); Formal analysis (equal); Funding acquisition (equal); Investigation (equal); Methodology (equal); Resources (equal); Validation (equal); Visualization (supporting); Writing‐original draft (lead); Writing‐review & editing (equal). **Paul Cryan:** Conceptualization (equal); Data curation (equal); Funding acquisition (equal); Investigation (equal); Methodology (equal); Project administration (equal); Resources (equal); Software (equal); Supervision (equal); Validation (equal); Visualization (equal); Writing‐review & editing (equal). **Marcos Gorresen:** Conceptualization (equal); Data curation (equal); Formal analysis (equal); Methodology (equal); Software (equal); Validation (equal); Visualization (lead); Writing‐review & editing (equal). **Lee Jay Fingersh:** Conceptualization (equal); Methodology (equal); Project administration (equal); Resources (equal); Writing‐review & editing (equal).

## ETHICAL APPROVAL

This study was entirely observational of natural bat behavior, and therefore, no ethics approval was required.

## Data Availability

The dataset and accompanying metadata are available on Dryad: https://doi.org/10.5061/dryad.q83bk3jh3.

## References

[ece37388-bib-0001] Abrahms, B. , Seidel, D. P. , Dougherty, E. , Hazen, E. L. , Bograd, S. J. , Wilson, A. M. , McNutt, J. W. , Costa, D. P. , Blake, S. , Brashares, J. S. , & Getz, W. M. (2017). Suite of simple metrics reveals common movement syndromes across vertebrate taxa. Movement Ecology, 5, 12. 10.1186/s40462-017-0104-2 28580149 PMC5452391

[ece37388-bib-0002] Andrews, R. D. , Pitman, R. L. , & Ballance, L. T. (2008). Satellite tracking reveals distinct movement patterns for Type B and Type C killer whales in the southern Ross Sea, Antarctica. Polar Biology, 31, 1461–1468. 10.1007/s00300-008-0487-z

[ece37388-bib-0003] Arnett, E. B. , & Baerwald, E. F. (2013). Impacts of wind energy development on bats: implications for conservation. In: R. R. Adams , S. Pedersen , & J. B. Shaw (Eds.), Bat Evolution, Ecology, and Conservation. New York: Springer Science + Business Media. 10.1007/978-1-4614-7397-8

[ece37388-bib-0004] Arnett, E. B. , Brown, W. K. , Erickson, W. P. , Fiedler, J. K. , Hamilton, B. L. , Henry, T. H. , Jain, A. , Johnson, G. D. , Kerns, J. , Koford, R. R. , Nicholson, C. P. , O’Connell, T. J. , Piorkowski, M. D. , & Tankersley, R. D. (2008). Patterns of bat fatalities at wind energy facilities in North America. Journal of Wildlife Management, 72, 61–78. 10.2193/2007-221

[ece37388-bib-0005] Arnett, E. B. , Huso, M. M. P. , Schirmacher, M. R. , & Hayes, J. P. (2011). Altering turbine speed reduces bat mortality at wind‐energy facilities. Frontiers in Ecology and the Environment, 9, 209–214. 10.1890/100103

[ece37388-bib-0006] Arnett, E. B. , & May, R. F. (2016). Mitigating wind energy impacts on wildlife: Approaches for multiple taxa. Human‐Wildlife Interactions, 10, 28–41.

[ece37388-bib-0007] Baerwald, E. F. , & Barclay, R. M. R. (2011). Patterns of activity and fatality of migratory bats at a wind facility in Alberta, Canada. Journal of Wildlife Management, 75, 1103–1114. 10.1002/jwmg.147

[ece37388-bib-0008] Barclay, R. M. R. , & Harder, L. D. (2003). Life histories of bats: Life in the slow lane. In T. H. Kunz , & S. Parsons (Eds.), Bat ecology (pp. 209–253). The University of Chicago Press.

[ece37388-bib-0009] Bennett, V. J. , Hale, A. M. , & Williams, D. A. (2017). When the excrement hits the fan: Fecal surveys reveal species‐specific bat activity at wind turbines. Mammalian Biology, 87, 125–129. 10.1016/j.mambio.2017.08.003

[ece37388-bib-0010] Cryan, P. M. (2003). Seasonal distribution of migratory tree bats (*Lasiurus* and *Lasionycteris*) in North America. Journal of Mammalogy, 84(2), 579–593.

[ece37388-bib-0011] Cryan, P. M. (2008). Mating behavior as a possible cause of bat fatalities at wind turbines. Journal of Wildlife Management, 72, 845–849. 10.1016/j.cub.2008.06.029

[ece37388-bib-0012] Cryan, P. M. , & Barclay, R. M. R. (2009). Causes of bat fatalities at wind turbines: Hypothesis and predictions. Journal of Mammalogy, 90, 1330–1340. 10.1644/09-MAMM-S-076R1.1

[ece37388-bib-0013] Cryan, P. M. , & Brown, A. C. (2007). Migration of bats past a remote island offers clues toward the problem of bat fatalities at wind turbines. Biological Conservation, 139, 1–11. 10.1016/j.biocon.2007.05.019

[ece37388-bib-0014] Cryan, P. M. , Gorresen, P. M. , Hein, C. D. , Schirmacher, M. R. , Diehl, R. H. , Huso, M. M. , Hayman, D. T. S. , Fricker, P. D. , Bonaccorso, F. J. , Johnson, D. H. , Heist, K. , & Dalton, D. C. (2014). Behavior of bats at wind turbines. Proceedings of the National Academy of Sciences, 111, 15126–15131. 10.1073/pnas.1406672111 PMC4210316 25267628

[ece37388-bib-0016] Erkert, H. G. (1982). Ecological aspects of bat activity rhythms. In T. H. Kunz (Ed.), Ecology of Bats (pp. 201–242). Plenum Press.

[ece37388-bib-0017] Fleming, T. H. 2019. Bat Migration. In: J. C. Choe (Ed.). Encyclopedia of Animal Behavior (2nd Edition) (pp. 3, 605–610). Cambridge, Massachusetts: Academic Press. 10.1016/B978-0-12-809633-8.20764-4

[ece37388-bib-0018] Foo, C. F. , Bennett, V. J. , Hale, A. M. , Korstian, J. M. , Schildt, A. J. , & Williams, D. A. (2017). Increasing evidence that bats actively forage at wind turbines. PeerJ, 5, e3985. 10.7717/peerj.3985 29114441 PMC5672837

[ece37388-bib-0019] Frick, W. F. , Baerwald, E. F. , Pollock, J. F. , Barclay, R. M. R. , Szymanski, J. A. , Weller, T. J. , Russell, A. L. , Loeb, S. C. , Medellin, R. A. , & McGuire, L. P. (2017). Fatalities at wind turbines may threaten population viability of a migratory bat. Biological Conservation, Biological Conservation, 209, 172–177. 10.1016/j.biocon.2017.02.023

[ece37388-bib-0020] Gibson, L. , Wilman, E. N. , & Laurance, W. F. (2017). How green is ‘Green’ Energy? Trends in Ecology & Evolution, 32, 922–935. 10.1016/j.tree.2017.09.007 29074270

[ece37388-bib-0021] Grodsky, S. M. , Jennelle, C. S. , Drake, D. , & Virzi, T. (2012). Bat mortality at a wind‐energy facility in southeastern Wisconsin. Wildlife Society Bulletin, 36, 773–783. 10.1002/wsb.191

[ece37388-bib-0022] Hartig, F. 2020. DHARMa: Residual Diagnostics for Hierarchical (Multi‐Level/Mixed) Regression Models. R package version 0.3.2.0. http://florianhartig.github.io/DHARMa/

[ece37388-bib-0023] Horn, J. W. , Arnett, E. B. , & Kunz, T. H. (2008). Behavioral responses of bats to operating wind turbines. Journal of Wildlife Management, 72, 123–132. 10.2193/2006-465

[ece37388-bib-0024] Hu, G. , Lim, K. S. , Horvitz, N. , Clark, S. J. , Reynolds, D. R. , Sapir, N. , & Chapman, J. W. (2016). Mass seasonal bioflows of high‐flying insect migrants. Science, 354, 1584–1587. 10.1126/science.aah4379. 28008067

[ece37388-bib-0025] Huzzen, B. E. , Hale, E. M. , & Bennett, V. J. (2020). An effective survey method for studying volant species activity and behavior at tall structures. PeerJ, 8, e8438. 10.7717/peerj.8438 32095329 PMC7023825

[ece37388-bib-0026] Jain, A. A. , Koford, R. R. , Hancock, A. W. , & Zenner, G. G. (2011). Bat mortality and activity at a northern Iowa wind resource area. The American Midland Naturalist, 165, 185–200. 10.1674/0003-0031-165.1.185

[ece37388-bib-0027] Jameson, J. W. , & Willis, C. K. R. (2014). Activity of tree bats at anthropogenic tall structures: Implications for mortality of bats at wind turbines. Animal Behaviour, 97, 145–152. 10.1016/j.anbehav.2014.09.003

[ece37388-bib-0028] Kunz, T. H. , Arnett, E. B. , Erickson, W. P. , Hoar, A. R. , Johnson, G. D. , Larkin, R. P. , Strickland, M. D. , Thresher, R. W. , & Tuttle, M. D. (2007). Ecological impacts of wind energy development on bats: Questions, research needs, and hypotheses. Frontiers in Ecology and the Environment, 5, 315–324.

[ece37388-bib-0029] Kunz, T. H. , Braun de Torrez, E. , Bauer, D. , Lobova, T. , & Fleming, T. H. (2011). Ecosystem services provided by bats. Annals of the New York Academy of Sciences, 1223, 1–38. 10.1111/j.1749-6632.2011.06004.x 21449963

[ece37388-bib-0030] Lindenberg, S. , Smith, B. , & O’Dell, K. (2008). 20% Wind energy by 2030. DOE/GO‐102008‐2567

[ece37388-bib-0031] Magnusson, A. , Skaug, H. J. , Nielsen, A. , Berg, C. W. , Kristensen, K. , Maechler, M. , van Bentham, K. J. , Bolker, B. M. , & Brooks, M. E. (2017). glmmTMB: generalized linear mixed models using Template Model Builder. R package version 0.1.3.

[ece37388-bib-0032] Mojica, E. K. , Watts, B. D. , & Turrin, C. L. (2016). Utilization probability map for migrating Bald Eagles in northeastern North America: A tool for siting wind energy facilities and other flight hazards. PLoS One, 11, e0157807. 10.1371/journal.pone.0157807 27336482 PMC4919076

[ece37388-bib-0033] Norberg, U. M. , & Rayner, J. V. M. (1987). Ecological morphology and flight in bats (Mammalia; Chiroptera): Wing adaptations, flight performance, foraging strategy and echolocation. Philosophical Transactions of the Royal Society of London B, 316, 335–427. 10.1098/rstb.1987.0030

[ece37388-bib-0034] Northrup, J. M. , & Wittemyer, G. (2013). Characterising the impacts of emerging energy development on wildlife, with an eye towards mitigation. Ecology Letters, 16, 112–125. 10.1111/ele.12009 23013218

[ece37388-bib-0035] O’Shea, T. J. , Cryan, P. M. , Hayman, D. T. S. , Plowright, R. K. , & Streicker, D. G. (2016). Multiple mortality events in bats: A global review. Mammal Review, 46, 175–190. 10.1111/mam.12064 29755179 PMC5942905

[ece37388-bib-0036] R Development Core Team , 2010. R: A language and environment for statistical computing. : R Foundation for Statistical Computing.

[ece37388-bib-0037] Reimer, J. P. , Baerwald, E. F. , & Barclay, R. M. R. (2010). Diet of hoary (*Lasiurus cinereus*) and silver‐haired (*Lasionycteris noctivagans*) bats while migrating through southwestern Alberta in late summer and autumn. American Midland Naturalist, 164(2), 230–237. 10.1674/0003-0031-164.2.230

[ece37388-bib-0038] Reimer, J. P. , Baerwald, E. F. , & Barclay, R. M. R. (2018). Echolocation activity of migratory bats at a wind energy facility: Testing the feeding‐attraction hypothesis to explain fatalities. Journal of Mammalogy, 99(6), 1472–1477. 10.1093/jmammal/gyy143

[ece37388-bib-0039] REN21 (2017). Renewables 2017: Global status report. Renewable and Sustainable Energy Reviews, 10.1016/j.rser.2016.09.082

[ece37388-bib-0040] Roeleke, M. , Blohm, T. , Kramer‐Schadt, S. , Yovel, Y. , & Voigt, C. C. (2016). Habitat use of bats in relation to wind turbines revealed by GPS tracking. Scientific Reports, 6, 28961. 10.1038/srep28961 27373219 PMC4931514

[ece37388-bib-0041] Rydell, J. , Bach, L. , Dubourg‐Savage, M.‐J. , Green, M. , Rodrigues, L. , & Hedenström, A. (2010a). Bat mortality at wind turbines in Northwestern Europe. Acta Chiropterologica, 12, 261–274. 10.3161/150811010X537846

[ece37388-bib-0042] Rydell, J. , Bach, L. , Dubourg‐Savage, M. J. , Green, M. , Rodrigues, L. , & Hedenström, A. (2010b). Mortality of bats at wind turbines links to nocturnal insect migration? European Journal of Wildlife Research, 56, 823–827. 10.1007/s10344-010-0444-3

[ece37388-bib-0043] Rydell, J. , Bogdanowicz, W. , Boonman, A. , Pettersson, S. , Suchecka, E. , & Pomorski, J. J. (2016). Bats may eat diurnal flies that rest on wind turbines. Mammalian Biology, 81(3), 331–339. 10.1016/j.mambio.2016.01.005

[ece37388-bib-0044] Satterfield, D. Z. , Sillett, T. S. , Chapman, J. W. , Altizer, S. , & Marra, P. P. (2020). Seasonal insect migrations: Massive, influential, and overlooked. Frontiers in Ecology and the Environment, 18(6), 335–344. 10.1002/fee.2217

[ece37388-bib-0045] Schlaepfer, M. A. , Runge, M. C. , & Sherman, P. W. (2002). Ecological and evolutionary traps. Trends in Ecology and Evolution, 17, 474–480. 10.1016/S0169-5347(02)02580-6

[ece37388-bib-0046] USDOE (U.S. Department of Energy) (2015). Wind Vision: A New Era for Wind Energy in the United States (Executive Summary, Full Report, and Appendices). U.S. Department of Energy (DOE), NREL (National Renewable Energy Laboratory), United States. https://www.nrel.gov/docs/fy15osti/63197‐2.pdf

[ece37388-bib-0047] Valdez, E. W. , & Cryan, P. M. (2013). Insect prey eaten by hoary bats (*Lasiurus cinereus*) prior to fatal collisions with wind turbines. Western North American Naturalist, 74, 516–524. 10.3398/064.073.0404

[ece37388-bib-0048] Voigt, C. C. , Lehnert, L. S. , Petersons, G. , Adorf, F. , & Bach, L. (2015). Wildlife and renewable energy: German politics cross migratory bats. European Journal of Wildlife Research, 61, 213–219. 10.1007/s10344-015-0903-y

[ece37388-bib-0049] Voigt, C. C. , Popa‐Lisseanu, A. G. , Niermann, I. , & Kramer‐Schadt, S. (2012). The catchment area of wind farms for European bats: A plea for international regulations. Biological Conservation, 153, 80–86. 10.1016/j.biocon.2012.04.027

[ece37388-bib-0050] Weller, T. J. , Castle, K. T. , Liechti, F. , Hein, C. D. , Schirmacher, M. R. , & Cryan, P. M. (2016). First direct evidence of long‐distance seasonal movements and hibernation in a migratory bat. Scientific Reports, 6, 34585. 10.1038/srep34585 27698492 PMC5048302

